# Some Further Evidence of the Efficiency of Sulphuric Ether as an Anæsthetic

**Published:** 1863-08

**Authors:** F. D. Lente

**Affiliations:** Surgeon to West Point Foundry


Some Further Evidence of the Efficiency of Sulphuric Ether as an Anæsthetic.—*By F. D. Lente, M.D., Surgeon to West Point Foundry.*—About a year ago, in one of the July numbers of this Journal, I furnished a list of surgical operations in a military hospital of which I was for a time surgeon-in-charge, in which sulphuric ether was administered by me, or under my direction, noting the *time* and *quantity* requisite for *complete* anæsthesia, with the object of inducing other surgeons to substitute this agent for *chloroform*, by proving conclusively, as these cases do, that the only objection which is now urged against ether (the length of time and the large quantity requisite) is entirely invalid, and due only to a faulty mode of administration. These cases were witnessed by many eminent surgeons from different parts of the country, who had been always in the habit of using chloroform, and by several army surgeons, who expressed great surprise and pleasure at the facility with which the patients were brought under the influence of the ether, and the simplicity of the means. One of them, at least, has since succeeded in procuring the substitution of ether for chloroform in a large public institution, of which he is one of the attending physicians.

Convinced myself years ago of the facility with which patients may be brought under the influence of sulphuric ether, and, with ordinary care, of its entire immunity from danger, I felt the importance of bringing forward occasionally fresh facts in illustration of this, and have accordingly done so at different times and in various journals. A number of private letters, received from some of the most distinguished members of the profession, expressive of their gratification at these eforts, and urging a continuance of the same, as well as a sense of duty, has induced me to solicit the attentive consideration of those surgeons especially who urge the above objections against sulphuric ether, to the following tabular statement of such operations as have occurred in my practice here since the publication of the cases above alluded to, in which I had occasion to use an anæsthetic, and in which there was opportunity for carefully noting the time and quantity.

| No. | Nature of Operation. | Time. | Quantity of Ether. | Sex. |
|---|---|---|---|---|
| | | Min. | Drachms. | |
| 1 | Extraction of Teeth - - - - - - | 3 | 12 | Fem. |
| 2 | " - - - - - - - - | 4 30 | 14 | " |
| 3 | Partial Amputation of Hand - - - - - | 4 | 16 | Male. |
| 4 | Extraction of Teeth - - - - - - - | 2 30 | 12 | Fem. |
| 5 | " - - - - - - - | 3 | 12 | " |
| 6 | Partial Amputation of Hand - - - - - - | 3 | 12 | Male. |
| 7 | Exsection of small Tumor - - - - - | 3 | 2 | Fem. |
| 8 | Extraction of Teeth - - - - - - - | 2 | 3 | " |
| 9 | " - - - - - - - | 3 | 8 | " |
| 10 | Deep Incision for Palmar Abscess - - - - - | Not noted. | 7 | Male. |
| 11 | Removal of Sequestrum of Humerus - - - - | 2 | 7 | " |
| 12 | Excessive Irritability of Bladder—Introduction of Catheter | 3 | 6 | Fem. |
| 13 | Excision of Cancerous Mamma - - - - - | 4 | 10 | " |
| 14 | Instrumental Delivery - - - - - - - | 3 | 10 | " |
| 15 | Reduction of Dislocation of Ankle Joint - - | 3 30 | 12 | Male. |
| 16 | For Necrosis of Jaw - - - - - - - | 4 | 12 | " |
| 17 | Diseased Bladder (Child)—Introduction of Catheter - | 1 30 | 1½ | " |
| 18 | Amputation of Finger - - - - - | 2 | 16 | " |
| 19 | Extraction of Teeth - - - - - - | 6 30 | 16 | Fem. |
| 20 | Amputation of Leg - - - - - - - | 4 | 12 | Male. |
| 21 | Cheiloplastic Operation - - - - - - - | 3 30 | 12 | " |
| 22 | Removal of Tumor from Neck - - - - - | 1 40 | 8 | Fem. |
| 23 | Incision of Carbuncle - - - - - - | 3 | 8 | Male. |
| 24 | Incision of Deep Palmar Abscess (Child) - - - | 1 30 | 5 | " |
| 25 | Amputation of Arm - - - - - - - | 2 | 8 | " |
| 26 | Removal of Neuromatous Tumor - - - - - | 4 30 | 8 | Fem. |
| 27 | Reduction of Old Dislocation of Elbow - - - | 4 | 9 | Male. |
| 28 | Extraction of Teeth - - - - - - - - | 4 30 | 8 | Fem. |
| 29 | " - - - - - - - | 2 30 | 7 | " |
| 30 | Reduction of Dislocation of Semilunar Cartilage - - | 5 | 14 | Male. |
| 31 | Amputation of Arm - - - - - - - | 2 | 3 | " |
| 32 | Crushed Foot—Partial Amputation - - - - | 2 | 6 | " |
| 33 | Amputation of Thigh - - - - - - | 2 | 4 | " |

Quite a number of the operations, it will be perceived, consists in the extration of teeth. In many cases from six to fourteen teeth were extracted during the etherization, the patient being all the while perfectly insensible. These operations afford a better test, perhaps, of the efficiency of an anæsthetic, than almost any other, as the patient is in an unfavorable posture, and the muscles must be perfectly relaxed in order that the operation may proceed satisfactorily. It will be noticed, accordingly, that a larger amount of ether, and a longer time, are required in these operations than in those of greater gravity, or those more strictly surgical.

I would also remark, that these operations have not been selected because in them the action of the anæsthetic was more satisfactory than in others, for such is not the fact, other equally painful operations having been equally successful as to the anæsthæsia during the same period, but because there was opportunity to note carefully the time and quantity, operations in the country not always allowing this.

The 1st, 3d, 6th, 7th, and 31st operations were performed by my partner, Dr. P. C. Barker. Dr. Davis, the dentist of this place, was generally the only professional witness of the dental operations, which were mostly performed by him; some of them by Mr. Saunders, hospital steward of West Point. Drs. P. C. Barker and William Young, of this place, were present at most of the operations, and noted the time and quantity in many instances. Dr. Sheldon, U. S. A., was present at the 11th, and Dr. Pryor, house-surgeon of Bellevue Hospital, at the 20th and 21st.

As regards the inhaler, I formerly used a cup-shaped sponge, large enough to cover the face, enveloping this with several folded towels; but one made with coarse and stiff towels alone, which are generally obtainable anywhere, is decidedly preferable, and it was this which was used in the above cases. The towels are to be laid together, and folded so as to make a cone sufficiently large to cover the nose and mouth; a handkerchief or soft cloth is then to be thrust into the apex of the cone, on which the ether is to be poured. I will describe again the mode of giving it, so as to insure the saving of time and ether; by using it more lavishly the average time might be diminished still more. A drachm or two (for an adult) are first poured rapidly into the cone, which is then approached close to the face of the patient (who has been previously directed to breathe through the nose and mouth, shutting the eyes). If coughing be excited, a little air is allowed to enter with the vapor of the ether for a few moments; two or three drachms more are then thrown quickly into the cone, which is rapidly replaced, and now kept closely applied to the face at every point; frequently an additional towel is thrown over it, if the vapor seems to escape. When the patient struggles, after becoming excited, and endeavors to tear the cone from the face, it must the more resolutely be kept there, and no air allowed to enter around the edges. Sometimes the breath is held for a few moments, in which case a little air may be allowed until he breathes freely again. The pulse should always be attended to, and if this be done, no fears regarding the ether need be entertained, as no sudden effect, like that often produced by chloroform, can be produced by it. A certain amount of practice and some tact are required to get the full effect of ether with an ounce or less, and within three minutes, unless the patient is very feeble or suffering from shock. One great cause of failure lies in withdrawing the inhaler too long from the face, from time to time, in order to replenish the ether, or for other causes, and not keeping it closely applied, thus cutting off all access of air except what enters through the pores of the towels; also in allowing a draught of air in the room; even in warm weather the apertures of the room should be closed during the first inhalation. I hope I may be excused for taking up space with these minutiæ, but without attention to them failure will often result.—*American Medical Times*, Aug. 29, 1863.

COLD SPRING, N. Y., Aug. 13, 1863.

